# Quantifying Uncertainty Due to Stochastic Weather Generators in Climate Change Impact Studies

**DOI:** 10.1038/s41598-019-45745-4

**Published:** 2019-06-25

**Authors:** Fosco M. Vesely, Livia Paleari, Ermes Movedi, Gianni Bellocchi, Roberto Confalonieri

**Affiliations:** 1 University of Milan, ESP, Cassandra Lab, via Celoria 2, 20133 Milan, Italy; 2 UCA, INRA, VetAgro Sup, Unité Mixte deRecherche sur Écosystème Prairial (UREP), Site de Crouel 5, Chemin de Beaulieu, 63000 Clermont, Ferrand France

**Keywords:** Projection and prediction, Environmental impact

## Abstract

Climate change studies involve complex processes translating coarse climate change projections in locally meaningful terms. We analysed the behaviour of weather generators while downscaling precipitation and air temperature data. With multiple climate indices and alternative weather generators, we directly quantified the uncertainty associated with using weather generators when site specific downscaling is performed. We extracted the influence of weather generators on climate variability at local scale and the uncertainty that could affect impact assessment. For that, we first designed the downscaling experiments with three weather generators (CLIMAK, LARS-WG, WeaGETS) to interpret future projections. Then we assessed the impacts of estimated changes of precipitation and air temperature for a sample of 15 sites worldwide using a rice yield model and an extended set of climate metrics. We demonstrated that the choice of a weather generator in the downscaling process may have a higher impact on crop yield estimates than the climate scenario adopted. Should they be confirmed, these results would indicate that widely accepted outcomes of climate change studies using this downscaling technique need reconsideration.

## Introduction

Climate change impact studies are affected by the statistical properties of the local weather time series^1^. Shifts in temperature and precipitation distributions, including extremes, are critically important for the analysis of agricultural^2^ and biological systems that incorporate complex, non-linear interactions at the soil-plant-atmosphere interface^3,4^. To develop climate scenarios, multi-model ensembles of General Circulation Models (GCMs) are used, which define the uncertainty in projections resulting from structural differences in the GCMs, as well as uncertainties in variations of initial conditions or parameterizations^5,6^. Future projections are based on alternative Representative Concentration Pathways (RCPs), each of them describing a potential future greenhouse gas concentration trajectory during the 21st century^7^. However, the direct use of climate predictions from GCMs is problematic because their coarse spatial resolution may result in biases and uncertainties at a local scale^6^, since GCMs that are used to project future climate scenarios provide gridded-area average simulations while the occurrence and intensity of extreme events strongly depend on local factors^8–11^. Stochastic weather generators (WGs) are routinely integrated into ecological or agro-meteorological studies to extend or interpolate incomplete weather data series for analyzing the extent of climate impacts within simulation applications, for instance with hydrological^12^, runoff^13,14^, ecosystem^15,16^, and crop yield models^2,17^. After calibration of site-specific parameters based on local weather data, WGs simulate synthetic daily weather time-series that are statistically similar to inputs^18–21^. In climate change studies, WGs are used to explore the effect of long-term changes in mean climate variables as well as changes in climatic variability and the frequency of extreme events^22^. Applications of WGs can also be found to downscale^23^ values of precipitation and air temperature with coarse spatio-temporal resolution as simulated from GCMs, based on probability density functions^24–26^. By empirically associating local-scale variables with large-scale atmospheric variables produced by GCMs, WG-based downscaling techniques underpin studies on regional and local-scale impact assessments. With weather generation, changes to climate obtained from GCM × RCP combinations are employed to alter the generator parameters for the site baseline to generate synthetic daily weather data for the future^27,28^. Weather generation has the advantage of being faster to compute than dynamic downscaling approaches such as Regional Climate Models that are driven by GCM outputs^29,30^.

The objective of this study was to assess the uncertainty introduced by WG-based downscaling when applied to projections of future climate. The analysis targeted scenarios centered on 2040 derived from two GCMs and two RCPs as well as the baseline period for multiple sites worldwide. As it is recommended to make multi-comparison studies using different WGs^31^, we compared three stochastic WGs: LARS-WG^32^, WeaGETS^33^ and CLIMAK^34^. The choice of the WGs was based on software accessibility and usability, as well as on the availability of complete documentation. The generated weather series were characterized using an extended set of weather indices and were used to feed a simulation model for rice yield estimates under baseline climate and future projections. Among available crops, rice was selected because of its importance as staple food for more than half of the world’s population.

## Results

This section first reports on the generated data and rice yield estimates obtained with a crop model, followed by an analysis of integrated climate indices calculated on both reference baseline and generated series. In the last part of the section, we address the effect of using alternative WGs on a set of climate indices by applying rank-based ANOVA to assess sources of uncertainty in the generation of downscaled future climate projections.

### Generated climate data

**Figure 1:**
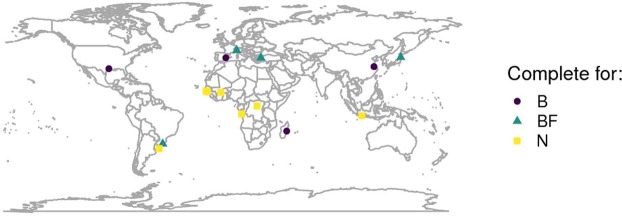
Sites involved in the study, marked according to their completeness for baseline and/or future projections. B, BF, N: sites with complete generated weather series for, respectively, baseline, baseline and future scenarios, none of them. The latter identifies sites where failures in generation occurred for some combinations WG × GCM × RCP.

Ensembles of baseline and future climate data (from four GCM × RCP combinations) were downscaled with three alternative WGs in mutiple sites worldwide (Fig. 1). LARS-WG returned complete data series in each situation, whereas CLIMAK and WeaGETS did not generate data for some sites and for some GCM × RCP combinations. The analysis of CLIMAK results also revealed the occurrence of some abnormal numerical values in weather series that were thus not included in further analysis.

### Effect of the generation of climate change projections on future rice yield estimates

**Figure 2:**
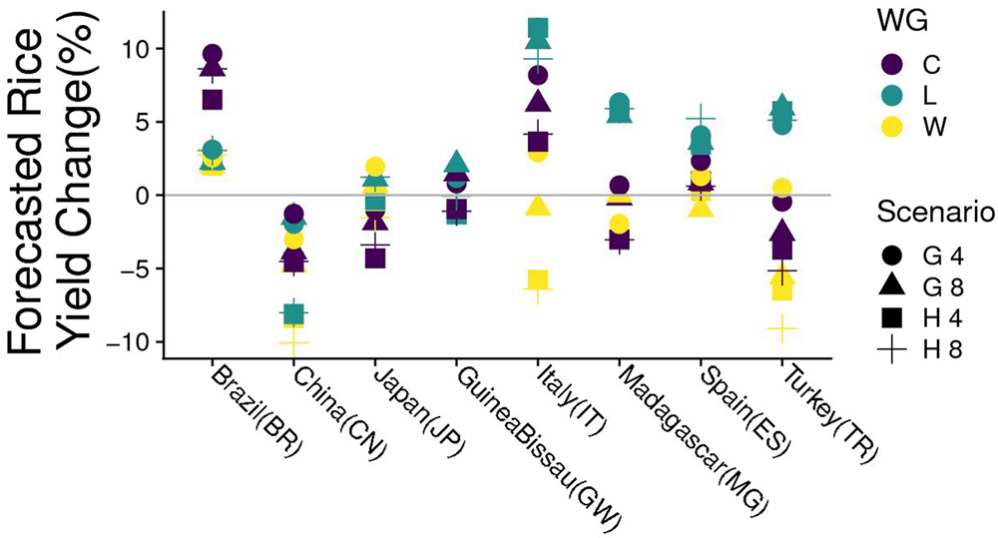
Climate change impact on rice yields (% compared to the baseline) estimated with the model WARM for different combinations weather generator (WG) × representative concentration pathway (RCP) × general circulation model (GCM) at eight sites distributed worldwide. sites are also labelled according to ISO 3166 standard. WGs are CLIMAK (C), LARS-WG (L), and WeaGETS (W). Scenarios indicate combinations of two GCMs, HadGEM2-ES (H) and GISS-ES-R (G), and two RCPs, RCP4.5 (4) and RCP8.5 (8).

The ranges of variation in projected rice yields (percentage changes in future scenarios with respect to the baseline) differed widely across sites (Fig. 2). For instance, distinct increases in rice yield were projected at the Brazilian site with all scenarios and WGs, whereas an opposite response was found in China but a more complex pattern was visible in other sites. With few exceptions, responses from different scenarios tended to cluster around the same WG; the same behaviour is advisable in aggregate aridity and climate classification indices (Supplementary Information). This indicates that overall variability due to alternate WGs (different colors in Fig. 2) may be larger than that induced by GCM × RCP combinations (different shapes in Fig. 2). The study case in Italy shows that rank reversal might also occur in rice yield projections when different WGs are used. For instance, either positive (with LARS-WG) or negative (with WeaGETS) impact was projected using HadGEM2-ES × RCP8.5 scenario. With different WGs, changes in projected impacts on rice yield was about ±5%. This is not a trivial value if for a big rice producer like China^35^ the energy contained in ±5% national rice production would correspond to 12800 megatons.

### Generated versus reference weather series

**Figure 3:**
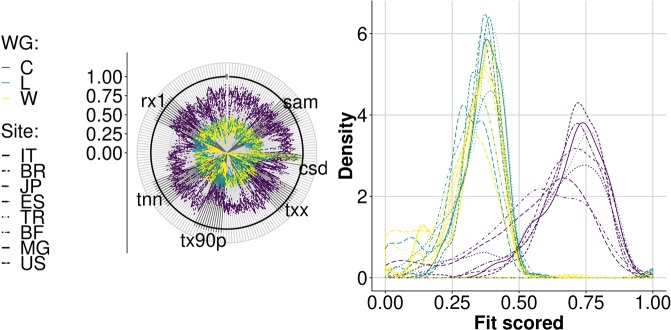
Agreement between generated and reference baselines. Line colors indicate weather generators (WG; C: CLIMAK; L: LARS-WG; W: WeaGETS), whereas line style corresponds to sites. (**a**) Overall fit of weather series for each index (one monthly index for each axis with code labels as reported in Supplementary Information); solid black lines indicate perfect fit to reference data. (**b**) Distribution density of the fit of all the indices analyzed (the closer to one, the more the whole set of reference distributions is reproduced by the WGs).

The analysis of generated weather series revealed the ability of each WG to reproduce the distribution of the values of an extended list of climate indices (reported in Supplementary Information) calculated on the reference series. The polar graph of Fig. 3a represents the multivariate system created with the set of indices used, with indices plotted on each radial axis. In particular, the value plotted on each axis represents the probability for reference and generated series to be the same for that index. The closer the contour lines are to one (thick black circle in Fig. 3a), the more similar the probability density functions for the two populations. Therefore, for each index, values close to one indicate a good agreement between generated and reference distributions. Figure 3 shows how the WGs differed for their likelihood to reproduce the most-probable value of each index (peaks in Fig. 3b). Some indices scored poorly regardless of the generator and the site (probably in relation to peculiarities inherent to reference weather series). However, while WeaGETS and LARS-WG offered a relatively more stable performance across the sites, the series generated by CLIMAK were often the most similar to reference ones.

**Figure 4:**
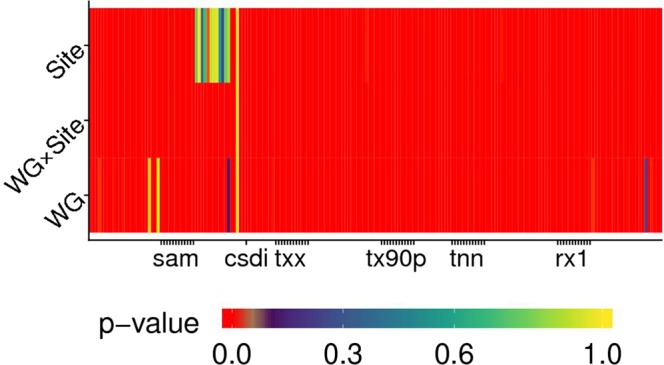
*p*-values for sources of variation affecting the difference between climate indices calculated on reference and generated baselines. *WG* refers to weather generators, *Site* to the location for which indices are calculated, *WG* × *Site* to their interaction. Red colour indicates significance (*p* < 0.05).

Results from a robust rank-based ANOVA-like method (factors being *WG* and *Site*) are presented in Fig. 4. Most of the indices were significantly affected (*p* < 0.05) by both *WG* and *Site*. Their interaction was also often significant (*p* < 0.05, indicated with red in Fig. 4). *Site* frequently resulted as the most probable source of differences rather than *WG*. However, for some indices (mainly those based on percentiles, since they are sensitive to extreme values, like tx90p - percentage of days having *T*_*max*_ > 90^th^ percentile), *WG* explained most of the variability observed. Some indices based on maximum and minimum daily air temperatures were often more affected by the *WG* than by the *Site*. For temperature-based indices, this was observded for monthly minimum of daily minima (TNN), as well as for monthly maximum of daily maxima (TXX).

### Uncertainty in downscaling future climate projections due to weather generators

**Figure 5:**
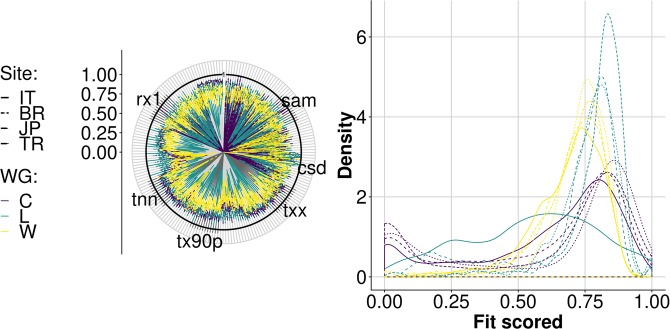
Probability to have the same density distribution for different climate indices. Color lines indicate weather generators (C: CLIMAK; L: LARS-WG; W: WeaGETS) whereas line styles correspond to sites (ISO 3166 labels). (**a**) Overlapping areas of probability density distributions of different monthly-based climate indices (radial axes, labelled in Supplementary Information) derived from different WGs for each Site × GCM × RCP combination. Solid black line indicates perfect fit of different combination GCM × RCP generated by the same WG for the same Site. The closer the lines to one, the smaller the difference between GCM × RCP combinations. In (**b**) Overall density similarity across indices for GCM × RCP combinations at each site using different generators.

**Figure 6:**
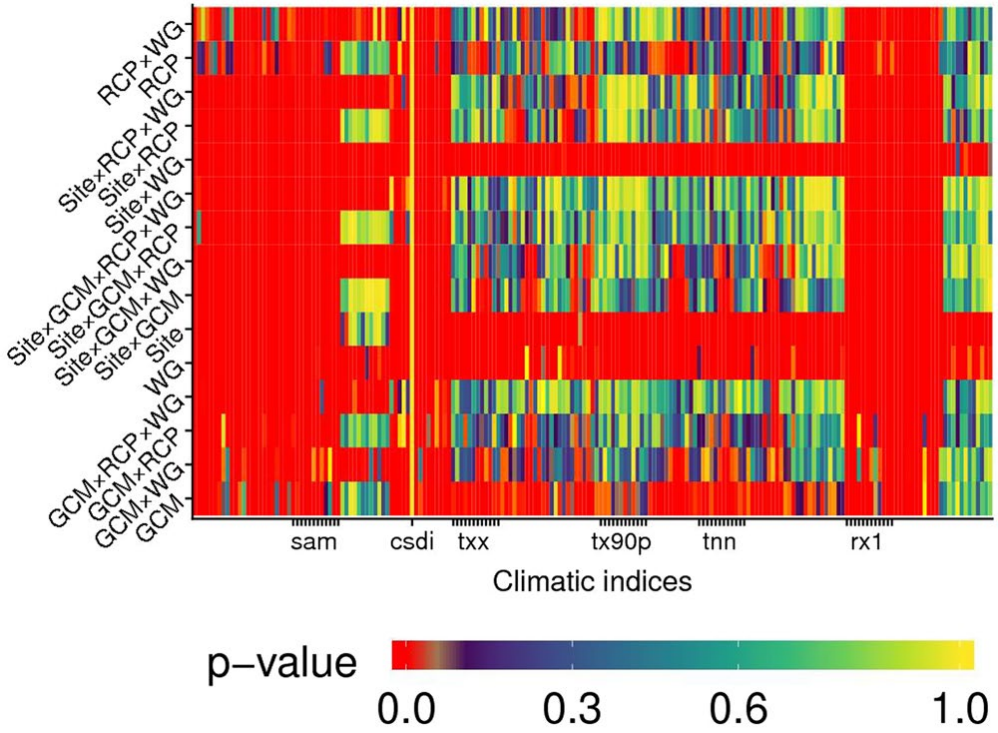
*p*-value for factors affecting the indices (described in Supplementary Information) derived from the analysis of generated future projections. Color scale is the same as in Fig. 4, with red indicating significant values (*p* < 0.05). *Site*: location of series; *RCP*: representative concentration pathway; *GCM*: general circulation model; *WG*: weather generator.

For each climate index, the proximity of different GCM × RCP combinations was assessed by approximating the area of overlap of the probability density distributions of the index values obtained with each WG. The results summarized in Fig. 5a,b return a quite high likelihood (0.7) to obtain the same value for different indices regardless of the GCM × RCP combination. This likelihood was indeed higher than the average resemblance of generated to reference baselines (<0.5). While a strong dependence of results on geographical location (site-specific) was somewhat expected, the most interesting outcome (Fig. 5b) was that results from different GCMs and RCPs tended to cluster by WG (more than by site). Given the influence of the different WGs on climate scenarios, the additional uncertainty that may result from the interaction between generation (WGs) and projections (GCMs and RCPs) was analyzed. A series of nonparametric robust rank order ANOVA tests was thus conducted to assess the effect of different factors (*Site*, *WG*, *GCM*, *RCP*, and their interactions) on each climate index calculated on future series (Fig. 6).

Two main patterns arose: some indices were strongly dependent from all the driving factors (red columns in Fig. 6), whereas some factors affected most of the indices (red rows). Besides, some indices, like the cold spell duration index (CSDI), were not significantly driven (*p* > 0.05) by any factor. Evapotranspiration- and rain-related indices (i.e., SAM and rx1day) were the most affected by the factors considered (as highlighted by thick red columns). Some factors, i.e., *WG*, *Site* × *WG*, *Site* and *GCM* (red rows), appeared to be the most influential for the majority of climate indices. Overall, *GCM* was the least significant factor, its average *p*-value being about 150% larger than for *Site* and about one order of magnitude larger than for *WG*. Results also indicated that influential factors are – to a certain extent – uniformly significant (*p* < 0.05) across different indices.

## Discussion

In climate change impact studies, the envelope of multiple GCM × RCP realizations includes the uncertainty accumulated throughout the process of climate generation^36,37^. At the end of the process, fine-scale climate data to feed impact models are developed from a variety of coarse scale GCMs and in dependence on alternative emission and socio-economic pathways. Uncertainties in model parameters, and imbalances in model equations, may propagate through the climate modelling chain, and these uncertainties may be enhanced or compensated during downscaling^38^ and then translated into impact estimates^39^. Studies analysing climate change have mostly emphasized the sources of uncertainty associated with adopting alternative GCM × RCP configurations in an ensemble of climate scenarios, but without fully considering the amount of uncertainty associated with using WG-based downscaling. Our results not only indicate that the impact of stochastic generation on precipitation and air temperature patterns within statistical downscaling methods can be substantial, but that the projected results may even be reversed, that is, the most favourable scenario obtained with a WG may become the worst with an alternative WG (and *vice versa*). This means that generated weather series may differ more due to the chosen stochastic generator rather than to the envelope of assumptions. Projection uncertainties handled by comparing different GCMs and RCPs could therefore be superseded by the uncertainties due to the WGs employed for the downscaling of climate variables. In our study, the array of climate indices and the rice yield analysis performed indicate an effect depending more on the WGs than on other factors (followed by the site dependant effect). While the effect of applying generated weather series in different areas, and for different crops and objectives is still to be assessed, further simulation experiments will verify if some biological resilience attenuate the impacts of stochastic weather generation on (simulated) biophysical processes.

While framing the uncertainty arising from using multiple WGs in the downscaling process, our study points out the (risk of) high dependence of climate change impact calculations on WGs (beyond the physics underlying the GCMs and the socio-economic scenarios driving the RCPs). Our results stress the need of verification of the appropriateness of several climate change studies using WG for downscaling, whose uncertainty - on the basis of what we observed - could be larger than expected. The application of our procedure to downscaled climate projections may provide information on the characteristics of uncertainty in local climate projections. In the context of agriculture and food security, this would consist of an ensemble of WGs, compared across an ensemble of crop models in a variety of agricultural regions. While there may be no best overall WG, the breadth of stochastic generation analysis can be used to provide end users with indication of the variability related to the stochastic weather generation. In conclusion, our findings advocate incorporation of alternative WG-based downscaling techniques as routine tools for the multi-ensemble methodology that already uses alternative RCPs/GCMs in impact studies. This would facilitate a move towards a complete assessment of uncertainty in simulations.

## Methods

### Site specific climate data

For the baseline period 1986–2005, we used the datasets of daily precipitation and air temperature available at 0.25° resolution from the Meteorological Archival and Retrieval System (MARS), which is the main repository of meteorological data at the European Centre for Medium-Range Weather Forecasts (ECMWF)^40^. To ensure a wide coverage while keeping the number of cases to a reasonable level, 15 sites (Fig. 1) were selected purposely from rice-growing areas.

### Performance evaluation of weather generators

In each site, the stochastic WGs LARS-WG^32^, WeaGETS^33^ and CLIMAK^34^ were used to reproduce the baseline weather series (1986–2005). Although the WGs used in this study are only a subset of those available, we think that the three WG types considered here are reasonably representative of current approaches to stochastic generation. The realism of stochastically generated precipitation and air temperature time-series with respect to the reference baseline was checked and portrayed with an array of 33 climatic metrics. They include 27 metrics defined by the Expert Team on Climate Change Detection and Indices/Climate Research Division (ETCCDI/CRD)^41^ complemented with a set of agro-meteorological metrics (SPEI^42^, de Martonne-Gottman aridity^43^, aridity index proposed by United Nation Environment Programme (UNEP) and adopted by Food and Agricolture Organization (FAO)^44^, Hargreaves Evapotraspiration^45^, Water Balance and Simple Aridity Measure^46^). The calculation of climatic and agro-climatic metrics helped to synthesize the information conveyed by alternative weather sources. For each metric, we first calculated site-specific cumulative distributions (exceedance probability) for the reference baseline and for the baselines estimated with each WG. Then, we integrated the overlapping area of two empirical cumulative distributions (with probability equal to one in case of perfect overlap and zero in the absence of any overlap). Third, matching the values of overlapping areas, we got the overall distribution for the whole set of metrics. In this way, one indicates perfect overlap for all the metrics. The second and third steps were repeated for the comparison of downscaled future climate projections. The effect of weather generation in the downscaling process was assessed based on the impact of downscaled climate change scenarios on rice yield estimates by using the output of a crop simulator (WARM^47^) as is commonly done in climate change impact studies.

### Downscaled future projections

In order to estimate a range of possible climates, we used two GCMs (GISS-E2-R^48^ and HadGEM2-ES^49^) that provided future projections centered on 2040 from RCP4.5 (moderate greenhouse gas concentration trajectory) and RCP8.5 (very high greenhouse concentration trajectory).

The fully coupled atmosphere-ocean GCM GISS-E2-R, developed by the National Aeronautics and Space Administration’s (NASA) Goddard Institute for Space Studies (GISS), is a contributor to the Coupled Model Intercomparison Project Phase 5^50^. With horizontal resolution of 2° latitude by 2.5° longitude and 40 vertical layers, the atmospheric model extends through the mesosphere (model top of 0.1 hPa). The latter is coupled to the Russell ocean model^51^, which has horizontal resolution of 1° latitude by 1.25° longitude and 32 vertical layers. As well, HadGEM2-ES is a coupled Earth System Model, used by the Met Office Hadley Centre for the Coupled Model Intercomparison Project Phase 5 centennial simulations. It comprises an atmospheric GCM at 1.25° (lat) × 1.875° (lon) horizontal and 40 km vertical resolutions, and an ocean GCM with a 1° horizontal resolution (increasing to 1*/*3° at the equator) and 40 vertical layers.

The delta-change technique perturbs the locally generated weather record with additive or multiplicative coefficients, based on the mean change from baseline to future frames extracted from the climate models. This approach circumvents the inherent limitations of a conventional delta-change approach that barely applies mean additive or multiplicative adjustments directly to reference baseline^26^.

### Climate change impact on estimated rice yields

As climate-change impact measure, we considered the projected to current rice yields estimated with the crop model WARM^47^ (averaged on 19-year long series, that is, 19 rice-growing seasons in 20 years of weather data in both hemispheres). We applied the impact study on rice yield at multiple sites using weather series obtained from different GCM × RCP × WG combinations. With three WGs, we could in particular estimate the relative weight of the uncertainties in the output with respect to the WG used.

### Variance analysis and decomposition

Robust rank analysis of variance (ANOVA) tests^52^ were conducted using climatic and agro-climatic indices coming from downscaled outputs under conditions identified by four factors. A split-plot design with *Site* as main plot, and *GCM* and *RCP* as sub- and sub-sub-plots was further extended to accommodate a fourth factor - weather generator (CLIMAK, LARS-WG, WeaGETS) - through additional subdivision of each sub-sub-plot into sub-sub-subplot. For the ANOVA, years were used as replication within each 20-year period. Here, the ANOVA tests were based on Rfit code^53^. Though the nonparametric ANOVA adopted does not require the assumptions of homogeneity of variance between the groups and the normality of residuals, a balanced design (the same amount of index values for each level of each factor) is required to detect the differences between the groups. Flaws in the generation process ended in having a limited set of series fulfilling the requirements (Fig. 1 and Supplementary Table 2). The reason is that the failure in one RCP × GCM × WG combination drives to failure for the whole location when trying to compare future projections. When one site encountered failures only in future scenarios, it was used in the analysis performed on the baseline (Fig. 4). Vice versa, when errors occurred only in the baseline generation (Fig. 6), series were only included in the analysis of future scenarios. In this way, all the ANOVA tests were performed according to a balanced design, depending on the occurrence of failures during weather generation.

## Supplementary information

SUPPLEMENTARY INFORMATION TO QUANTIFYING UNCERTAINTY DUE TO STOCHASTIC WEATHER GENERATORS IN CLIMATE CHANGE IMPACT STUDIES

## Data Availability

The datasets generated during and/or analysed during the current study are available from the corresponding author on reasonable request. ECMWF policies prompt not to reproduce, distribute, license, transfer, assign, sell, disclose or otherwise forward the Archive Products. By the way, weather data may come from whatever source with no difference in the considered terms. Anyway http://apps.ecmwf.int/datasets allows to access some datasets. CLIMAK official page for further information and availability: http://semola.uniud.it/index.php?id=129\L=1. LARS-WG home page: http://resources.rothamsted.ac.uk/mas-models/larswg. WeaGETS host page on MathWorks: https://it.mathworks.com/matlabcentral/fileexchange/29136-stochastic-weather-generator–weagets. WARM official web page: http://www.cassandralab.com/applications/2.
