# Supplementary material for: Quantifying Uncertainty Due to Stochastic Weather Generators in Climate Change Impact Studies
Source: Sci Rep. 2019 Jun 25;9:9258. doi: 10.1038/s41598-019-45745-4 (PMC6592885; doi:10.1038/s41598-019-45745-4)
Supplement: Supplementary file 1 — SUPPLEMENTARY INFORMATION TO QUANTIFYING UNCERTAINTY DUE TO STOCHASTIC WEATHER GENERATORS IN CLIMATE CHANGE IMPACT STUDIES [file 41598_2019_45745_MOESM1_ESM.docx]

QUANTIFYING UNCERTAINTY DUE TO STOCHASTIC WEATHER GENERATORS IN CLIMATE CHANGE IMPACT STUDIES

FOSCO M. VESELY*^A^*^∗^

LIVIA PALEARI*^A^*

ERMES MOVEDI*^A^*

*University of Milan, ESP, Cassandra Lab, via Celoria 2, 20133, Milan, Italy*

GIANNI BELLOCCHI*^B^*

*UCA, INRA, VetAgro Sup, Unité Mixte deRecherche sur Écosystème Prairial (UREP), Site de Crouel 5, Chemin de Beaulieu, 63000 Clermont-Ferrand, France*

ROBERTO CONFALONIERI*^A^*^∗^

*University of Milan, ESP, Cassandra Lab, via Celoria 2, 20133, Milan, Italy*

## Indices index

The list of adopted indices and monthly scaled code name follows; the sorting is the same adopted in all indices listings in the article. Most of them are from the set collected by ETCCDI integrated by some agro-climatic indices. Monthly-based indices are considered as standalone on their relative period and arranged on seasons in order to be comparable across different grid point. Description of ETCCDI refers to [http://etccdi.pacificclimate.org/list](http://etccdi.pacificclimate.org/list_27_indices.shtml) [27](http://etccdi.pacificclimate.org/list_27_indices.shtml) [indices.shtml](http://etccdi.pacificclimate.org/list_27_indices.shtml).[1]

**a-l:EThg:** Hargreaves evapotraspiration calculated using SPEI package [2].

**m-x:Bal:** water balance. Obtained as: *Rain* − *EThg* (mm).

**m:CDD:** Maximum length of dry spell, maximum number of consecutive days with RR *<* 1 mm: Let *RR_ij_* be the daily precipitation amount on day *i* in period *j*. Count the largest number of consecutive days where: *RR_ij_ <* 1 mm.

**y-aj: SAM:** Synthetic AgroMeteorological indicator [3] calculated as $SAM=\frac{Rain-ET0}{Rain+ET0}$

**ak-av:SPEI:** Standardized precipitation evapotranspiration index [4] calculated using the software SPEI [2].

**aw:SU:** Number of summer days: Annual count of days when TX (daily maximum temperature) *>* 25°. Let *TX_ij_* be daily maximum temperature on day *i* in year *j*. Count the number of days where: *TX_ij_ >* 25°.

**ax:ID:** Number of icing days: Annual count of days when TX (daily maximum temperature) *<* 0°. Let *TX_ij_* be daily maximum temperature on day *i* in year *j*. Count the number of days where: *TX_ij_ <* 0°.

**ay:WSDI:** Warm spell duration index: Annual count of days with at least six consecutive days when TX *>* 90^th^ percentile Let *Tx_ij_* be the daily maximum temperature on day *i* in period *j* and let Txin90 be the calendar day 90^th^ percentile centered on a 5-day window for the base period 1961-1990. Then the number of days per period is summed where, in intervals of at least six consecutive days: *TX_ij_ > Txin*90.

**az:FD:** Number of frost days: Annual count of days when Tn (daily minimum temperature) *<* 0°. Let *Tn_ij_* be daily minimum temperature on day *i* in year *j*. Count the number of days where: *Tn_ij_ <* 0°.

**ba:TR:** Number of tropical nights: Annual count of days when Tn (daily minimum temperature) *>* 20°. Let *Tn_ij_* be daily minimum temperature on day *i* in year *j*. Count the number of days where: *Tnij >* 20°.

**bb:CSDI:** Cold speel duration index: Annual count of days with at least six consecutive days when TN < 10^th^ percentile. Let *Tn_ij_* be the daily maximum temperature on day *i* in period *j* and let *TNin10* be the calendar day 10^th^ percentile centered on a 5-day window for the base period 1961-1990. Then the number of days per period is summed where, in intervals of at least six consecutive days: *Tn_ij_ < TNin*10.

**bc:SDII:** Simple precipitation intensity index: Let *RR_wj_* be the daily precipitation amount on wet days, w (*RR* ≥ 1 mm) in period *j*. If *W* represents number of wet days in *j*, then:
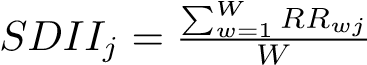
.

**bd:R10mm:** Annual count of days when *PRCP* ≥ 10 mm: Let *RR_ij_* be the daily precipitation amount on day *i* in period *j*. Count the number of days where: *RR_ij_* ≥ 10 mm**.**

**be:R20mm:** Annual count of days when *PRCP* ≥ 20 mm. Let *RR_ij_* be the daily precipitation amount on day *i* in period *j*. Count the number of days where: *RR_ij_* ≥ 20 mm**.**

**bf:Rnnmm:** Annual count of days when *PRCP* ≥ *nn* mm, *nn* being a user-defined threshold. Let *RR_ij_* be the daily precipitation amount on day *i* in period *j*. Count the number of days where: *RR_ij_* ≥ *nn* mm.

**bg:CDD:** Maximum length of dry spell, maximum number of consecutive days with *RR* *<* 1 mm. Let *RR_ij_* be the daily precipitation amount on day *i* in period *j*. Count the largest number of consecutive days where: *RR_ij_ <* 1 mm.

**bh:CWD:** Maximum length of wet spell, maximum number of consecutive days with *RR* ≥ 1 mm. Let *RR_ij_* be the daily precipitation amount on day *i* in period *j*. Count the largest number of consecutive days where: *RR_ij_* ≥ 1 mm.

**bi:R95pTOT:** Annual total *PRCP* when *RR* *>* 95^th^ percentile. Let *RR_wj_* be the daily precipitation amount on a wet day *w* (*RR* ≥ 1 mm) in period *i* and let *RR_wn_*95 be the 95^th^ percentile of precipitation on wet days in the 1961-1990 period. If *W* represents the number of wet days in the period, then:
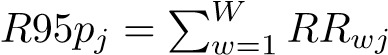
, where *RR_wj_* ≥ *RR_wn_*95.

**bj:R99pTOT:** Annual total *PRCP* when *RR* *>* 99^th^ percentile. Let *RR_wj_* be the daily precipitation amount on a wet day *w* (*RR* ≥ 1 mm) in period *i* and let *RR_wn_*99 be the 99^th^ percentile of precipitation on wet days in the 1961-1990 period. If *W* represents the number of wet days in the period, then:
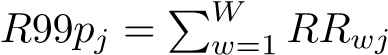
, where *RR_wj_* ≥ *RR_wn_*99.

**bk:PRCPTOT:** Annual total precipitation in wet days: Let *RR_ij_* be the daily precipitation amount on day *i* in period *j*. If *I* represents the number of days in *j*, then
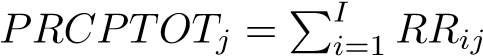
.

**bl:bw-TXx:** Monthly maximum value of daily maximum temperature: Let *TXx_kj_* be the daily maximum temperatures in month *k*, period *j*. The maximum daily maximum temperature each month is then: *TXx_kj_* = *max*(*TXx_kj_*).

**Bx:ci-TXn:** Monthly minimum value of daily maximum temperature: Let *TXn* be the daily maximum temperatures in month *k*, period *j*. The minimum daily maximum temperature each month is then: *TXn_kj_* = *min*(*TXn_kj_*).

**cj:cu-TX10p:** Percentage of days when *TX* *<* 10^th^ percentile. Let *TX_ij_* be the daily maximum temperature on day *i* in period *j* and let *TXin10* be the calendar day 10^th^ percentile centered on a 5-day window for the base period 1961-1990. The percentage of time for the base period is determined where: *TX_ij_ < TXin10*. To avoid possible inhomogeneity across the in-base and out-base periods, the calculation for the base period (1961-1990) requires the use of a bootstrap processure [5].

**cv:dg-TX90p:** Percentage of days when *TX* *>* 90^th^ percentile. Let *TX_ij_* be the daily maximum temperature on day *i* in period *j* and let *TXin90* be the calendar day 90^th^ percentile centered on a 5-day window for the base period 1961-1990. The percentage of time for the base period is determined where *TXij > TXin90*. To avoid possible inhomogeneity across the in-base and out-base periods, the calculation for the base period (1961-1990) requires the use of a bootstrap processure [5].

**dh:ds-TNx:** Monthly maximum value of daily minimum temperature. Let *TNx_kj_* be the daily minimum temperatures in month *k*, period *j*. The maximum daily minimum temperature each month is then: *TNx_kj_* = *max*(*TNx_kj_*).

**dt:ee-TNn:** Monthly minimum value of daily minimum temperature. Let *TNn_kj_* be the daily minimum temperatures in month *k*, period *j*. The minimum daily minimum temperature each month is then: *TNn_kj_* = *min*(*TNn_kj_*).

**ef:eq-TN10p:** Percentage of days when *TN* *<* 10^th^ percentile. Let *TN_ij_* be the daily minimum temperature on day *i* in period *j* and let *TNin10* be the calendar day 10^th^ percentile centered on a 5-day window for the base period 1961-1990. The percentage of time for the base period is determined where *TN_ij_* *< TNin10*. To avoid possible inhomogeneity across the in-base and out-base periods, the calculation for the base period (1961-1990) requires the use of a bootstrap processure [5].

**er:fc-TN90p:** Percentage of days when *TN* *>* 90^th^ percentile. Let *TN_ij_* be the daily minimum temperature on day *i* in period *j* and let *TNin90* be the calendar day 90^th^ percentile centered on a 5-day window for the base period 1961-1990. The percentage of time for the base period is determined where *TN_ij_* *> TNin*90. To avoid possible inhomogeneity across the in-base and out-base periods, the calculation for the base period (1961-1990) requires the use of a bootstrap processure [5].

**fd:fo-Rx1day:** Monthly maximum 1-day precipitation. Let *RR_ij_* be the daily precipitation amount on day *i* in period *j*. The maximum 1-day value for period *j* are: *Rx1day_j_* = *max*(*RR_ij_*).

**fp:ga-Rx5day:** Monthly maximum consecutive 5-day precipitation. Let *RR_kj_* be the precipitation amount for the 5-day interval ending *k*, period *j*. Then maximum 5-day values for period *j* are: *Rx5day_j_* = *max*(*RR_kj_*).

**gb:gm-DTR:** Daily temperature range, monthly mean difference between *TX* and *TN*. Let *TX_ij_* and *TN_ij_* be the daily maximum and minimum temperature respectively on day i in period j. If *I* represents the number of days in *j*, then:
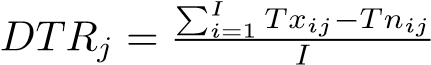


Table S1. Site coordinates (EPSG:4326)

| Site | Lat | Long |
| --- | --- | --- |
| Italy | 45*.*32 | 8*.*41 |
| China | 32*.*22 | 119*.*26 |
| Indonesia | −7*.*32 | 109*.*51 |
| Brazil | −29*.*98 | −51*.*37 |
| Japan | 39*.*68 | 140*.*95 |
| Spain | 39*.*35 | −0*.*39 |
| Turkey | 39*.*37 | 28*.*00 |
| Uruguay | −33*.*85 | −54*.*29 |
| Burkina Faso | 11*.*38 | −4*.*40 |
| Guinea Bissau | 11*.*75 | −15*.*35 |
| Madagascar | −19*.*77 | 48*.*77 |
| Texas | 30*.*55 | −94*.*90 |
| CongoN | 0*.*5 | 25 |
| CongoS | −5*.*9 | 12*.*33 |
| Senegal | 12*.*55 | −16*.*33 |

## Climate syntethic indices

Aridity indices mentioned in the text (and described here below) give differences up to 50% in some cases (fig. S1). On future climate scenarios, the differences between CLIMAK and WeaGETS (relative to LARS-WG) may result up to one order of magnitude (Fig. 3b). This means that alternative WGs portray a contrasting picture in terms of climate characterization at a given site.

# Impact on Climates


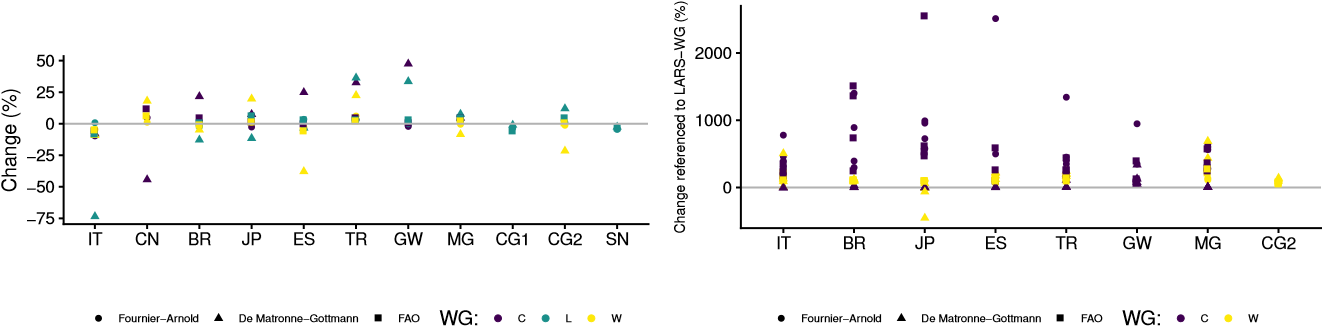


Figure S1. Weather generation effect on aridity indices at different grid points(ISO 3166 label). Weather generators (WG) refer to CLIMAK (C), LARS-WG (L), and WeaGETS W). Comparison between aridity indices computed on observed and generated baseline weather data.


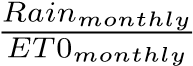
Aridity indices:

**FAO:** Suggested [6] as index for climate classification is calculated as *FAO* =

**De Martonne-Gottmann:** the index is widely used and calculated monthly as
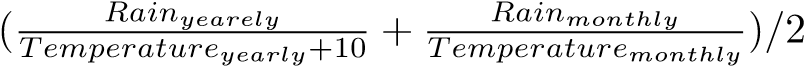


**Fourniern-Arnoldus:** explore the role of each month on yearly-scale rains amount.

The index is calculated as
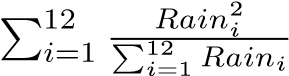


Table S2. Comparison of three weather generators at simulating daily precipitation occurrence and amount, and maximum and minimum daily temperatures (options used in this study are reported)

| Weather  Generator | Generation  Precipitation  Air temperature occurrence amount | | | Notes |
| --- | --- | --- | --- | --- |
| **CLIMAK** | Separate estimates for maximum and minimum daily values; different parameters for dry and rainy days; approach: second-order Fourier series | First-order Markov chain | Two-parameter Gamma distribution | It proved reliable in comparative studies (to catch extreme rain events), under a variety of conditions |
| **LARS-**  **WG** | Separate estimates for maximum and minimum daily values; different parameters for dry and rainy days; approach: third-order Fourier series | Semi-empirical distribution of dry and wet series (to overcome limitations of Markov chain-based approaches) | Semi-empirical precipitation distribution | Specifically designed for climate change impact studies, it is extensively used in diverse climates |
| **WeaGETS** First-order linear autoregressive model for both maximum and minimum daily values | | Third-order Markov chain | Two-parameter Gamma distribution | It brings together options of other generators into one package |

## WARM Configuration

Rice yield forecasts are based on WARM model execution. Due to differences in management, exploited varieties and growing conditions, crops are configured site-specifically as usually done for similar studies. Table S3 reports the set of parameters used for the simulations involved in estimation of the effect of the generation of climate change projections on future rice yield**.**

## References

1. For the Pacific Climate Impacts Consortium, D. B. *climdex.pcic: PCIC Implementation of Climdex Routines* R package version 1.1-6 (2015). [https://CRAN.R-project.org/package=climdex.pcic.](https://CRAN.R-project.org/package=climdex.pcic)
2. Santiago Beguería and Sergio M. Vicente-Serrano (2017). SPEI: Calculation of the Standardised Precipitation-Evapotranspiration Index. R package version 1.7. <https://CRAN.R-project.org/package=SPEI>
3. Confalonieri, R., Bregaglio, S., Acutis, M., 2010. A proposal of an indicator for quantifying model robustness based on the relationship between variability of errors and of explored conditions. Ecological Modelling, 221, 960-964.
4. Vicente-Serrano, S. M., Beguer´ıa, S. & L´opez-Moreno, J. I. A multiscalar drought index sensitive to global warming: the standardized precipitation evapotranspiration index. *Journal of Climate* **23,** 1696–1718 (2010).
5. Zhang, X., Hegerl, G., Zwiers, F. W. & Kenyon, J. Avoiding inhomogeneity in percentile-based indices of temperature extremes. *Journal of Climate* **18,** 1641–1651 (2005).
6. UNEP, 1993. World Atlas of Desertification. The United Nations Environment Programme (UNEP), London.
